# Book Review: Quorum Sensing vs. Quorum Quenching: A Battle With No End in Sight

**DOI:** 10.3389/fcimb.2018.00106

**Published:** 2018-04-04

**Authors:** Nisarg Gohil, Robert Ramírez-García, Happy Panchasara, Shreya Patel, Gargi Bhattacharjee, Vijai Singh

**Affiliations:** Synthetic Biology Laboratory, Department of Microbiology, School of Biological Sciences and Biotechnology, Institute of Advanced Research, Gandhinagar, India

**Keywords:** quorum sensing, quorum quenching, auto-inducers, multi-drug resistance, biosensors

The discovery of antibiotics to eliminate infectious diseases has been one of the most remarkable medical achievements at the beginning of twentieth century (Pina et al., 2010). However, within the past few decades, bacteria have rapidly co-evolved to become resistant against antibiotics through horizontal gene transfer or by mutation in target genes. These issues must be addressed, and that need is growing. Currently, the uncontrolled use of antibiotics has led us to the nascent era of antibiotic-resistant microorganisms, commonly known as multidrug-resistant (MDR) and extensive drug resistant (XDR) microorganisms. The WHO (World Health Organization) has reported a serious shortfall in the new antibiotics pipeline to defeat the growing threat of antimicrobial resistance, mainly because most of the drugs currently in clinical trials are modifications of existing antibiotics and are only short-term solutions (Antibacterial Agents in Clinical Development, 2017). The WHO has also published a list of “antibiotic-resistant priority pathogens"—a catalog of 12 families of bacteria divided into three categories. These are sorted upon the urgency of the need for new antibiotics as critical, high, and medium (Prioritization Pathogens Infographic, 2017), to guide and encourage research and development of new antibiotics.

An alternative approach is to control bacteria via quorum sensing (QS). It has been discovered that bacteria are able to communicate through signaling pheromones or auto-inducers by a system called QS, a specialized mechanism used to sense population density. Bacteria are able to sense their own population density and use it as a trigger to switch to virulent and pathogenic behaviors that can facilitate their survival. It has been essential to find better strategies to tackle this serious issue, which led to the discovery of QS quenchers or QS inhibitors (QSIs). QSIs inhibit QS of bacteria (e.g., through the inactivation of virulence factors) rather than killing them, which helps to decrease damage to commensal microbiota.

Whilst there is ample scientific literature published in the last few years, the book “Quorum Sensing vs. Quorum Quenching: A Battle with No End in Sight,” edited by Kalia (2015), has not only compiled the diverse pieces of literature but also provided in-depth knowledge of QS mechanisms and strategies to inhibit the QS. The unique selling points of this book are that the assembled chapters are written in an easy to follow manner, and it has been written by eminent scientists from around the world with expertise in the research area. The editor has meticulously divided the book into seven parts, comprising 31 chapters that focus on the following aspects: (i) QS mediated processes, (ii) QS systems in microbes, (iii) detectors for QS signals, (iv) natural QSIs, (v) synthetic QSIs, (vi) alternative strategies as QSIs, and (vii) biotechnological applications of QSIs.

The QS system plays a vital role in biofilm formation, bioluminescence, symbiosis, pigment production, antibiotic production, sporulation, motility, and in toxin and virulence factor production (Rutherford and Bassler, 2012; Chaudhari et al., 2014; Bassler, 2016). The first part (part I) of this book describes the evolution of MDRs, and the mechanisms involved in antibiotic resistance, including chromosomal mutation at the target site of antibiotics, increased efflux and reduced influx of antibiotics, enzymatic drug modification or degradation, and protection or alteration of the drug target. Similarly, it discusses the processes that drive the evolution of MDR, including horizontal gene transfer and mutational induction. It also presents the contributing factors that lead to its evolution, such as genetic, environmental (the dissemination of antibiotics and drugs into the environment by hospitals, research laboratories, and pharmaceutical industries), social, and other factors (underuse, overuse, misuse of antibiotics, and the lack of proper government policies) with examples as case studies.

In general, biofilm plays a key role in protection and for the enhanced attachment to different hosts, which allows efficient access to oxygen and nutrients. Several studies have proven that microorganisms that produce biofilm could be shown to be 1,000–1,500 times more resistance to antibiotics than otherwise (Chandki et al., 2011). A number of studies show how QS cascade mechanisms regulate biofilm formation in *Pseudomonas aeruginosa, Vibrio cholerae, Vibrio fischeri, Bacillus subtilis*, and *Staphylococcus aureus* in this part. The QS also regulates nitrogen fixation, competence, and sporulation, and these have been described in detail. The authors have given a special emphasis on QS mechanism that is involved in nitrogen fixation. There is also description of the toxins and virulence factors of the pathogens, and for better understanding the book gives details of their molecular mechanisms and regulation. This study can allow us to design a new diagnostic toolbox to identify and further combat infections. Finally, the authors have explained why the absolute configuration of auto-inducers and QSI is imperative in QS and quorum quenching.

Natural QS circuits have been identified in over 25 species of Gram-negative bacteria, by means of a LuxI/LuxR-type circuit (Miller and Bassler, 2001) and recently few synthetic QS circuits have been constructed (Hong et al., 2012; Hennig et al., 2015). There is a need to understand the molecular mechanism of organisms to inhibit their pathogenicity by generating novel anti-QS compounds or quorum quenchers. In the second part (part II) of this book, the authors have discussed QS molecular mechanisms in *Escherichia coli, Acinetobacter baumannii, Pseudomonas, Clostridia, Aeromonas, Enterococci*, and *Bacillus* spp.

A number of efforts to detect and monitor QS signaling molecules and autoinducers have been performed. Part III describes the different classes of available biosensors with different methods for the detection and monitoring of QS signals. Additionally, biosensor sensitivity and limitation have also been discussed, which can be used for the development of biosensing systems. Furthermore, this part highlights *Caenorhabditis elegans*, a non-mammalian *in vivo* model organism that shows around ~65% of human disease genes (Baumeister and Ge, 2002), helping to better understand host-pathogen interactions and also to study *in vivo* efficacy of the QSI compounds. It has presented itself to be easy to handle, economic, amenable, and genetically tractable worm with a rapid rate of reproduction. The recent advances using *C. elegans* are thoroughly emphasized here. In addition to this part shows various targets of QS systems for the prevention of bacterial virulence, including genes responsible for the production of QS autoinducers, signaling molecules by degradation or inactivation, receptors, and efflux pumps to block it.

Part IV highlights natural QSIs. An enzymatic QS inhibition shows a wider range of inhibitory potential by degrading or modifying auto-inducers, when compared to inhibitors that are generally target specific. The recent developments in enzymatic QS inhibition in bacteria, plants, nematode infection models, and in aquaculture fields are thoroughly described. The authors have also discussed QS inhibition aspects in fungi toward developing and discovering potent QSIs by giving outstanding examples such as farnesol, farnesoic acid from *Candida albicans*, patulin and penicillic acid (metabolites), cellulases, proteases, and amylases (enzymes) for QS inhibition and biofilm degradation. Marine organisms are also rich sources, providing thousands of biologically and chemically bioactive compounds. The anti-QS properties, the nature of their derived compounds, and their future are thoroughly mentioned here.

Similarly, synthetic small organic QS inhibitors also have the potential to interfere with the bacterial QS mechanism. Part V comprises these and similar ideas, and the main aim of this part is to provide a guideline to design and discover new QS antagonists for antibacterial therapeutic applications. It highlights the strategies and recent key discoveries that attempted to develop synthetic QS signal analog with excellent examples. This part also draws attention to the development of synthetic QSI against *Pseudomonas aeruginosa*, an opportunistic human pathogen that causes a variety of systemic infections through biofilm formation and virulence factors. There is discussion of the main three QS systems (*las, rhl, pqs*) of *P. aeruginosa*, and how they are interconnected. The possible solutions and therapeutic strategies to interrupt these systems pathways are well elucidated for overwhelming infection. In addition, the authors present two approaches (random screening from natural resources and rational drug design) in *Enterococcus faecalis* for the targeting the *fsr* system, which is a leading gene cluster responsible for regulation of pathogenicity-related proteases (gelatinase and serine proteases).

The next part (part VI) covers some of recent alternative strategies for QS inhibition. Firstly, pheromone-guided antimicrobial peptides and fusion peptides, which consist of a targeting domain to guide selective binding to target and a killing domain of a known antimicrobial peptide. The second is the synergetic effect of QSIs with antibiotics, which allows the reduction of bacterial resistance against drugs (Moussaoui and Alaoui, 2016) and nanotechnological approaches. Last but not least is the heterologous expression of QS inhibitory genes in diverse organisms. It also provides a list of the heterologous expression of acyl homoserine lactone (autoinducer of Gram-negative bacteria)-lactonase in QS systems of various organisms. Finally, part VII describes the potential applications of QSIs in various fields such as medical, agriculture, fisheries and aquacultures, and water treatments. Additionally, this part is also points out the biotechnological applications in aquacultures with detailed structural features of both chemical and biological QSIs, as well as their modes of action and their use.

Recent breakthroughs in targeted genome engineering by CRISPR-Cas9 (Jinek et al., 2012, 2013; Mali et al., 2013; Singh et al., 2017, 2018) has opened new avenues for genome editing and regulation in a wide range of organisms and cell types. In this light, enabling the use of CRISPR for the modification of bacterial genomes (Jiang et al., 2013) has opened the way toward the development of sequence-specific CRISPR-based antimicrobials (Beisel et al., 2014; Bikard et al., 2014; Bikard and Barrangou, 2017). In the future, we can believe that the CRISPR-Cas9 system may replace antibiotics entirely, and maybe as simply as by taking a single CRISPR pill.

Altogether, this book is a good attempt to address the problem of antibiotic drug resistance. It provides all the information about bacterial cell-cell communication systems and possible strategies to tackle pathogenic strains. This book is an excellent, informative, and unique scientific contribution showing how the battle between humans and bacteria continues as a medical and scientific arms race that continues for both. The editor has splendidly compiled all of the information for the scientific communities that serve mankind in this context. Aside from this, we found that the book is written in such a way that readers will tend to build more and more interest, being a compelling read that is easy to follow. This book is highly recommended to anybody with interest in microbiology, from students to researchers, since it provides with novel resolutions in this arms race against dangerous and deadly pathogens.

## Author contributions

NG, RR-G, HP, SP, GB, and VS have designed and written the manuscript. VS has supervised and finalized the final version of the manuscript.

### Conflict of interest statement

The authors declare that the research was conducted in the absence of any commercial or financial relationships that could be construed as a potential conflict of interest.
